# Exposure to Occupational Carcinogens and Non-Oncogene Addicted Phenotype in Lung Cancer: Results from a Real-Life Observational Study

**DOI:** 10.3390/cancers17182997

**Published:** 2025-09-13

**Authors:** Enrico Oddone, Luca D’Amato, Roberta Pernetti, Domenico Madeo, Luca Toschi, Sara Farinatti, Giulia Riva, Lucrezia Spina, Luigia Ferrante, Catharina Conde, Laura Deborah Locati, Federico Sottotetti, Franca Barbic

**Affiliations:** 1 Department of Public Health, Experimental and Forensic Medicine, University of Pavia, 27100 Pavia, Italy; luca.damato@unipv.it (L.D.); roberta.pernetti@unipv.it (R.P.); 2 Hospital Occupational Medicine Unit (UOOML), ICS Maugeri IRCCS, 27100 Pavia, Italy; domenico.madeo@icsmaugeri.it; 3 IRCCS Humanitas Research Hospital, Cancer Center, Rozzano, 20089 Milan, Italy; luca.toschi@cancercenter.humanitas.it (L.T.); sara.farinatti@cancercenter.humanitas.it (S.F.); 4 Medical Oncology Unit, ICS Maugeri IRCCS, 27100 Pavia, Italy; giulia.riva@icsmaugeri.it (G.R.); lucrezia.spina@icsmaugeri.it (L.S.); lauradeborah.locati@unipv.it (L.D.L.); federico.sottotetti@icsmaugeri.it (F.S.); 5 International Medical School, Humanitas University, Pieve Emanuele, 20072 Milan, Italy; luigia.ferrante@st.hunimed.eu (L.F.); catharina.conde@st.hunimed.eu (C.C.); 6 Department of Internal Medicine and Medical Therapy, University of Pavia, 27100 Pavia, Italy; 7 Department of Biomedical Sciences, Humanitas University, Pieve Emanuele, 20072 Milan, Italy; franca.barbic@hunimed.eu; 8 IRCCS Humanitas Research Hospital, Rozzano, 20089 Milan, Italy

**Keywords:** lung cancer, occupational exposures, phenotype, non-oncogene addicted

## Abstract

**Background/Objectives**: Lung cancer (LC) remains one of the most lethal malignancies worldwide, with both environmental and occupational exposures contributing to its incidence. While oncogene-addicted tumors—defined by single driver mutations—have garnered attention due to their therapeutic implications, less is known about the mutational landscape of tumors potentially arising from occupational exposure to carcinogens. This real-life observational study aimed to assess whether previous occupational exposure to lung carcinogens correlates with distinct LC phenotypes, particularly non-oncogene-addicted (nOA) profiles. **Methods**: A total of 199 LC patients were enrolled across two specialized oncology centers in Northern Italy between 2021 and 2023. Each participant underwent detailed occupational history taking and molecular characterization using next-generation sequencing. Patients were stratified into nonexposed (NE), low exposed (LE), and high exposed (HE) to carcinogens for lung based on standardized questionnaires and sector-specific assessments. **Results**: No significant differences were found in histological subtypes across exposure groups. However, people with adenocarcinoma and high occupational exposure to lung carcinogens were more frequently characterized by a nOA phenotype compared to those with low occupational exposure. Logistic regression models—adjusted for age, sex, and smoking habits—confirmed that HE patients had a significantly higher likelihood of developing nOA tumors (OR = 3.07; 95% CI: 1.16–8.11; *p* = 0.023). This association persisted after adjusting for smoking habits Exposures occurring 5–10 years before diagnosis seemed to be associated with an increased nOA profile. **Conclusions**: These findings suggest that high levels of exposure to occupational carcinogens impact LC phenotypes. Indeed, these phenotypes are more complex to treat and show the worst prognosis. Assessing the occupational exposure to lung carcinogens during work may offer prognostic insights and support the request for more adequate compensation for the patients. Further studies are warranted to validate these results and to explain the mechanisms that produce the differences observed in LC phenotypes in people with high exposure to occupational carcinogens.

## Simple Summary

Lung cancer remains one of the most aggressive cancers, often linked to smoking and environmental risk factors. However, less is known about how occupational exposure to carcinogens—such as those encountered in construction, manufacturing, or other industrial settings—may influence the type of lung cancer that develops. In this real-world study from Northern Italy, we examined 199 lung cancer patients and assessed their past exposure to occupational lung carcinogens. We found that people who worked in settings characterized by higher exposure to occupational carcinogens were more likely to develop a form of lung cancer classified as “non-oncogene-addicted” compared to people with lower occupational exposure. These results suggest that environmental exposure may play a role in mutational features of lung cancer. The patient’s work history may help predict cancer behavior and guide treatment strategies. This study also highlights the importance of future research into occupational health and cancer prevention.

## 1. Introduction

Malignant neoplasms represent one of the leading causes of mortality from non-communicable diseases in industrialized countries, with lung cancer (LC) accounting for approximately 23.5 deaths per 100,000 individuals in 2021 [1]. While hereditary factors explain only a minority of malignant tumors [2], the majority are associated with modifiable lifestyle determinants such as tobacco use, excessive alcohol intake, and exposure to environmental and occupational carcinogens [3,4].

Occupational exposure to carcinogens constitutes a considerable contributor to cancer incidence, with an estimated 17% of lung cancer-related deaths attributable to work-related exposures [5]. Nonetheless, occupational cancers remain markedly underreported worldwide [6,7,8,9], including in Italy [10].

The development of lung cancer is generally a protracted, multistep process characterized by the progressive accumulation of genetic mutations and epigenetic modifications that disrupt key cellular pathways [11]. Histologically, LC is categorized into small-cell lung cancer (SCLC) and non-small-cell lung cancer (NSCLC). SCLC, a hypermutated subtype, shows a strong association with tobacco smoking, whereas NSCLC—particularly adenocarcinoma—can also arise in nonsmokers or in individuals with poorly defined environmental risk factors.

Since the early 2000s, advances in molecular biology have facilitated the identification of driver mutations in NSCLC. These mutations, positively selected during tumor progression, confer a proliferative advantage to cancer cells and are encompassed within the concept of “oncogene addiction”. In Western countries, up to 60% of pulmonary adenocarcinomas are now considered oncogene-addicted tumors [12], most commonly involving alterations in genes such as EGFR, ALK, ROS1, and BRAF.

Next-generation sequencing (NGS) has become an essential diagnostic tool in lung adenocarcinoma, enabling the detection of clinically actionable mutations [13,14,15,16,17].

To date, to the best of our knowledge, no significant differences have been documented in clinical presentation, diagnostic approaches, or treatment strategies between lung cancers arising from occupational carcinogen exposure and those unrelated to occupational risk factors.

This study explores the hypothesis that prior occupational exposure to lung carcinogens, as defined by the International Agency for Research on Cancer (IARC), may shape the phenotypic and molecular characteristics of lung cancer. Specifically, we examined whether such exposures are associated with distinct histological subtypes and oncogenic pathways, including the presence of oncogene addiction. Confirmation of this association would highlight the clinical relevance of obtaining a detailed occupational history in lung cancer management and prognosis. Moreover, identifying patients whose lung cancer may be attributable to occupational exposures could inform compensation eligibility and strengthen epidemiological surveillance.

## 2. Materials and Methods

We conducted a real-world observational study involving patients diagnosed with lung cancer at two secondary care centers in Northern Italy: the Oncology Department of ICS Maugeri in Pavia and the Cancer Center of Humanitas Research Hospital in Milan (CC-HRH). All patients underwent comprehensive clinical evaluation and molecular profiling with next-generation sequencing (NGS) as part of routine care. Occupational exposure to established lung carcinogens was assessed using validated, ad hoc questionnaires. Tumor histology and mutational status were subsequently correlated with exposure history to investigate potential relationships between the external exposome and lung cancer phenotype. In addition, smoking history and its interaction with occupational exposure were systematically analyzed.

### 2.1. Study Population

This study included all consecutive patients diagnosed with lung cancer (LC) who were monitored or treated at ICS Maugeri and CC-HRH between October 2021 and November 2023. All patients presenting to the two centers during this period were considered eligible for enrollment, except for those who explicitly declined participation.

No exclusion criteria were applied with respect to histological subtype; therefore, the cohort comprised adenocarcinoma, squamous-cell carcinoma, small-cell lung cancer (SCLC), and other less common histological variants.

Comprehensive clinical, diagnostic, epidemiological, and therapeutic data were retrieved from medical records for each participant.

The study protocol was approved by the respective Local Ethics Committees (ICS Maugeri approval no. 2598/CE; CC-HRH approval no. 1459653/CE) and conducted in accordance with the principles of the Declaration of Helsinki.

### 2.2. Clinical and Anatomopathological Data: Classification of Non-Oncogene-Addicted (nOA) Cases

Clinical and anatomopathological data were collected from individual medical records. Disease staging was determined according to the TNM classification, 8th edition (UICC). Morphological and immunohistochemical features—including adenocarcinoma, squamous-cell carcinoma, and small-cell lung cancer (SCLC)—were assessed using standard clinical procedures.

In adenocarcinoma cases, specific gene alterations were analyzed using Easy PGX ready kits (Diatech Pharmacogenetics, Jesi, Italy) and real-time PCR for ALK, ROS1, RET, MET exon 14 skipping, KRAS, and BRAF. EGFR mutations (exons 18–21) were tested with either Easy PGX ready kits (Diatech Pharmacogenetics, Jesi, Italy) or IDYLLA (Biocartis, Mechelen, Belgium). When available, clinically relevant mutations were further characterized by next-generation sequencing (NGS) using the Oncomine Precision Assay GX (Genexus, Thermo Fisher, Waltham, MA, USA). Both EGFR and BRAF alterations were evaluated in tissue biopsies or liquid biopsies obtained from blood samples.

Oncogene addiction (OA) was defined as the presence of a single driver mutation, irrespective of the affected gene, with the exception of KRAS, which is generally regarded as a marker of environmental exposure [18]. KRAS mutations were not systematically assessed in patients from ICS Maugeri. Tumors without an actionable driver mutation were classified as non-oncogene-addicted (nOA).

For each case of LC, the date of diagnosis corresponded to that of the first histological report. The estimated onset of LC was calculated by applying a latency period of 13.6 years, consistent with the model proposed by Nadler and Zurbenko [19,20].

### 2.3. Occupational Exposure Evaluation

Data on occupational exposure and lifestyle habits were collected through a two-step process. First, a standardized questionnaire—recommended by the Occupational Cancer Study Group of the Lombardy Region (Italy)—was administered to obtain a preliminary assessment. Interviews were conducted by occupational physicians who were informed of the patients’ clinical status but blinded to their histological and molecular profiles. All interviews were carried out during hospitalization or day-hospital visits for therapy.

This first-level evaluation aimed to distinguish patients with potential occupational exposure to carcinogens from those likely unexposed, based on the industrial sector and job tasks reported. Occupational roles were coded according to the International Standard Industrial Classification of All Economic Activities (ISIC, Revision 4, 2008) [21].

In the second step, patients identified as potentially exposed to lung carcinogens completed a sector-specific questionnaire developed by the Italian National Insurance Institute for Work Accidents and Occupational Diseases (INAIL) within the ReNalOcCaM system [22]. Classification of exposures was based on the list of lung carcinogens defined by the International Agency for Research on Cancer (IARC) [23].

This phase allowed for a more detailed assessment of individual exposure to occupational lung carcinogens. A subject was classified as having high exposure if their longest-held occupation was in a sector where exposure to pulmonary carcinogens is considered highly probable based on current scientific evidence regarding the industrial setting and specific job tasks. For example, a worker with a 40-year career, of which 25 years were spent as a truck driver, was classified as highly exposed due to the prolonged exposure to diesel exhaust, which is recognized as a lung carcinogen by IARC. Conversely, a worker who spent 15 out of 40 years in an occupation with diesel exhaust exposure, while the remaining years were in jobs without exposure to pulmonary carcinogens, was classified as having low exposure. Individuals with no documented occupational exposure (e.g., administrative clerks) were classified as unexposed.

To strengthen and contextualize the classification methodology, we summarized in Tables S10 and S11 the available literature linking specific occupational sectors to an increased risk of lung cancer. Following both levels of assessment, patients were stratified into three subgroups: (a) higher exposed (HE), defined as those whose longest-held job was in a sector with established exposure to lung carcinogens; (b) lower exposed (LE), defined as those with at least one job involving potential exposure, although not representing the predominant occupation; and (c) not exposed (NE), defined as those without any reasonable occupational exposure to lung carcinogens.

Tables S10 and S11 in the Supplementary Materials detail, for each occupational sector, the carcinogenic agents considered, according to IARC classification. Asbestos exposure was also included, as in certain occupational settings it remains relevant despite the nationwide ban implemented in Italy in 1992 [24].

### 2.4. Smoking Exposure Assessment

Patients’ smoking habits were assessed by quantifying both smoking duration and cumulative exposure, expressed in pack-years. The questionnaire also investigated potential passive smoke exposure (yes/no), regardless of whether it occurred in the workplace or in a domestic setting. All data analyses were adjusted for active smoking exposure.

### 2.5. Statistical Analysis

Differences in demographic, clinical, and histological characteristics of lung cancer (LC), as well as occupational exposure profiles, between patients enrolled at the two participating centers were assessed using the χ^2^ test. Age differences were evaluated with either the independent-samples t-test or the Wilcoxon–Mann–Whitney test, depending on data distribution.

Logistic regression models were applied to examine the association between occupational exposure to lung carcinogens and oncogene-addicted (OA) status, as previously defined. Three models were developed: (a) Model 1, an unadjusted analysis of exposure classification (HE, LE, NE) and OA status; (b) Model 2, adjusted for potential confounders including smoking status (never, former, or current smoker at diagnosis), age at diagnosis (continuous), and sex; and (c) Model 3, adjusted for the same covariates as Model 2, with smoking history expressed as pack-years (calculated as the number of cigarettes smoked per day divided by 20 and multiplied by the years of active smoking). Model fit was evaluated using the Hosmer–Lemeshow test and standardized residuals (Table S4, Supplementary Materials). Unadjusted models demonstrated poor fit, whereas adjusted models—particularly those including age, smoking status, and sex—showed adequate fit. To assess multicollinearity among predictors, variance inflation factors (VIFs) were calculated for each independent variable (Table S5, Supplementary Materials).

Statistical significance was set at a two-sided *p* value < 0.05. All analyses were conducted using STATA software, version 18 (StataCorp LLC, College Station, TX, USA, 2024).

## 3. Results

A total of 199 patients were enrolled in the study, comprising 100 from ICS Maugeri and 99 from CC-HRH. Demographic characteristics and age at enrollment for the two cohorts are summarized in Table 1. The majority of lung cancer cases occurred in individuals aged over 60 years (*n* = 158; 79.4%), with no statistically significant differences observed between the two centers.

At the time of diagnosis, approximately 40% of patients were current smokers, whereas fewer than 10% had never smoked. The median pack-year value was slightly higher among patients from CC-HRH compared with those from ICS Maugeri; however, differences in smoking history between the two centers did not reach statistical significance, irrespective of the metric applied (Table 1).

In contrast, the proportion of patients classified as highly exposed (HE) to occupational lung carcinogens was significantly greater in the ICS Maugeri cohort. This difference reached statistical significance both when all histological subtypes were considered (*p* = 0.010, Table 1) and when the analysis was restricted to patients with adenocarcinoma (*p* = 0.013).

### 3.1. Smoking Exposure Assessment

No significant differences in smoking habits were observed among the groups (Table 1). The potential interaction between smoking status and occupational exposure is reported in Table S6 (Supplementary Materials), with no statistically significant associations detected. A sensitivity analysis, presented in Table S9 (Supplementary Materials), yielded consistent results.

The frequency distribution of passive smoking (yes/no) did not vary according to smoking habits or exposure classification. Similarly, when smoking history was reconstructed in terms of pack-years, the median values did not differ significantly between patients who reported passive smoke exposure and those who did not (Mann–Whitney test, *p* = 0.345). For these reasons, the passive smoking variable was not included in the adjustment of statistical estimates, and it is unlikely that this factor biased our findings.

### 3.2. Age at Diagnosis and Age at First Employment

Most patients (74.9%) were aged 60 years or older at the time of diagnosis (Table 2). The median age at first employment was significantly lower among patients classified as highly exposed (HE) compared with those with lower or no exposure, both in the overall cohort (16 vs. 19.5 years, *p* < 0.001) and within the adenocarcinoma subgroup (16 vs. 20 years, *p* < 0.001).

A significant difference was also observed in the decade of workforce entry: 51.1% of HE patients began employment before 1970, compared with 36.8% of LE and NE patients (*p* < 0.001). This trend remained significant when the analysis was restricted to adenocarcinoma cases (51.4% vs. 33.1%, *p* < 0.001).

Patients enrolled at CC-HRH were significantly younger than those from ICS Maugeri (median age 66 vs. 69 years, *p* = 0.03), a difference that persisted in the adenocarcinoma subgroup (65.5 vs. 69 years, *p* = 0.02).

Notably, a greater proportion of ICS Maugeri patients had entered the workforce before 1970 compared with CC-HRH patients (48.0% vs. 32.3%, respectively). A similar distribution was observed among adenocarcinoma patients (46.2% vs. 27.8%).

### 3.3. Tumor Histological Types and Gene Mutations

Adenocarcinoma was the most prevalent histological subtype among enrolled patients (Table 2). Squamous-cell carcinoma represented the second most frequent subtype, with 26 cases (13.1%). No statistically significant association was observed between occupational exposure to lung carcinogens and histological subtypes of lung cancer (Table S1).

The prevalence of gene mutations was comparable between the two study sites (Table 2). As expected, KRAS mutations were the most frequently detected. However, this mutation was not systematically assessed in patients from ICS Maugeri, which may account for the apparent differences in KRAS mutation frequency between cohorts (Table 2). For this reason, KRAS alterations were considered from a clinical rather than a statistical perspective, and patients with KRAS mutation alone were not classified as “oncogene-addicted”, given the frequent association of this mutation with environmental and/or occupational exposures.

The second most common alteration involved the EGFR gene, detected in 14 cases overall (7.0%), including 5 patients from ICS Maugeri and 9 from CC-HRH.

### 3.4. Occupational Exposures

Most patients were classified as NE (117 cases; 58.8% of the total cohort—49 from ICS Maugeri and 68 from CC-HRH). A smaller proportion (*n* = 34; 17.1%) were categorized as LE. Notably, the number of patients classified as HE was greater in the ICS Maugeri cohort (31 cases) compared with the CC-HRH cohort (16 cases).

**Figure 1:**
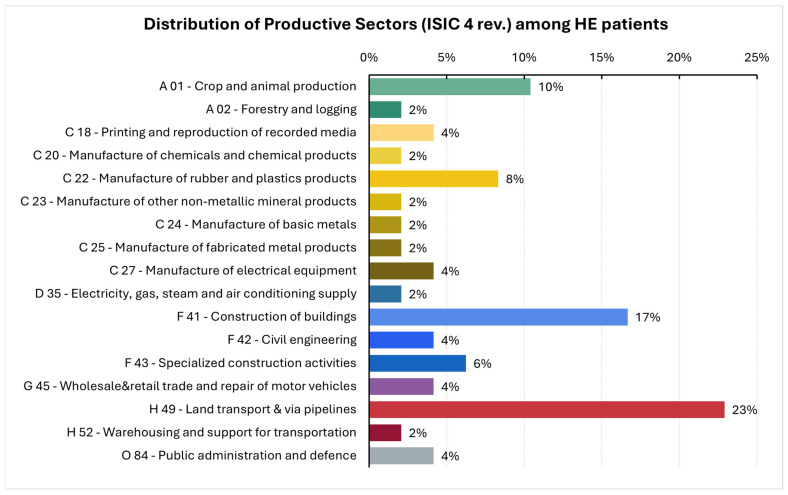
Distribution in percentage of productive sectors where the patients identified as highly exposed to lung carcinogens (HE) worked. The classification of HE was conducted also taking into consideration the specific job task performed during their longest period of activity (see text for detail). The productive sectors were classified following the International Standard Industrial Classification of All Economic Activities (ISIC, version 4, 2008) [21].

The occupational sectors of HE patients are summarized in Figure 1. The most represented sectors included land transportation, construction, and agriculture, in addition to several manufacturing industries, which together accounted for 24% of HE cases.

The most frequently identified occupational carcinogens among HE patients were diesel engine exhaust, ambient air pollution, rubber production by-products, welding fumes, asbestos, and bis(chloromethyl) ether.

Tables S10 and S11 (Supplementary Materials) provide detailed information on the occupational sectors and specific job tasks for each case classified as highly exposed. For each sector, we also summarized the available literature on exposure measurements and/or epidemiological evidence linking that sector to an increased risk of lung cancer (see references attached to Tables S10 and S11 for details).

### 3.5. Occupational Exposure and Oncogene-Addiction State

**Figure 2:**
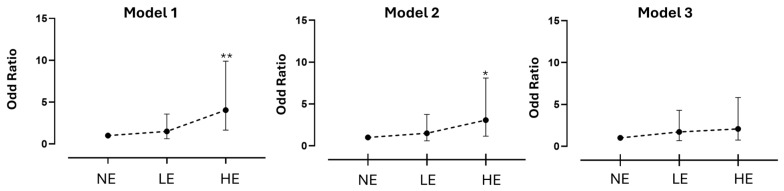
Odds ratios and corresponding 95% confidence intervals for non-oncogene-addicted lung adenocarcinoma phenotype corresponding to the group of patients classified as Never Exposed (NE), considered as a reference, Low Exposed (LE), and High Exposed (HE), as defined in the text. Model 1 (**left panel**) shows the unadjusted data, Model 2 (**middle panel**) shows data adjusted for sex, age at diagnosis, and smoking habits (never, former, and current smokers at diagnosis), and Model 3 shows data adjusted for sex, age at diagnosis, and smoking habits in cigarette pack-years. * *p* < 0.05; ** *p* < 0.01.

We observed a significantly increased likelihood of developing a non-oncogene-addicted (nOA) phenotype among HE patients compared with those NE. This association remained statistically significant after adjustment for age at diagnosis and smoking status. Specifically, in Model 2, the odds ratio (OR) was 3.07 (95% CI 1.16–8.11, *p* = 0.023). When further adjusted according to Model 3, the association persisted but did not reach conventional statistical significance (OR 2.08; 95% CI 0.74–5.82; *p* = 0.162) (Table 3, Figure 2).

Specifically, Logistic Regression Model 2 (adjusted for smoking status, age, and sex) yielded an OR of 3.07 (95% CI: 1.16–8.11; *p* = 0.023). Model 3, which additionally incorporated smoking intensity in pack-years, produced a lower but still elevated OR of 2.08 (95% CI: 0.74–5.82), although this result did not achieve statistical significance (*p* = 0.162) (Table 3, Figure 2).

Occupational exposure was evaluated at the estimated time of lung cancer onset, as well as 5 and 10 years before this point. Exposure status at diagnosis and at 5 and 10 years before diagnosis was also considered. No statistically significant differences were found between the two study cohorts across these time windows (Table S2). Nonetheless, although not meeting conventional significance thresholds, the results suggest a potential temporal correlation between occupational exposure and oncogenic effects. Workers exposed to lung carcinogens at least 5–10 years prior to diagnosis exhibited a 1.5- to 2.0-fold higher risk of developing nOA adenocarcinoma compared with unexposed individuals at the same time points (Table S3). Similarly, individuals occupationally exposed 5 or 10 years before estimated disease onset demonstrated a 1.4- to 1.8-fold increased risk of nOA adenocarcinoma. However, this association was no longer apparent after adjustment for smoking history expressed in pack-years (Model 3, Table S3).

## 4. Discussion

### 4.1. Actionable Gene Mutations, Histotypes, and Occupational Exposure

Our study showed that the distribution of actionable gene mutations differed between lung cancers in patients with occupational exposure to carcinogens and those without such exposure. The former appeared more frequently characterized by a non-oncogene-addicted (nOA) mutational profile.

Recent insights into the role of environmental factors—including occupational exposures—and their potential interactions in carcinogenesis [25] have heightened interest in exploring the relationship between exposure and mutational burden in lung cancer (LC). To our knowledge, no data are currently available on the potential influence of different environmental and occupational exposures on LC mutational load. In this context, generating evidence on the development of LC in individuals occupationally exposed to carcinogens, and characterizing their mutational profiles, may enhance our understanding of etiopathogenesis, elucidate mechanisms of tumorigenesis, and support evidence-based prevention strategies.

From a clinical perspective, metastatic LC remains a disease with an overall dismal prognosis across biological subtypes. Nevertheless, targeted therapies (e.g., EGFR inhibitors for EGFR-mutated LC) have consistently demonstrated meaningful clinical benefit in patients with oncogene-addicted tumors [26]. By contrast, tumors lacking oncogene addiction—seemingly more frequent among patients with occupational exposures—require alternative therapeutic approaches, primarily chemotherapy and/or immunotherapy. These treatments are associated with greater toxicity and less predictable efficacy [27].

We currently lack a definitive explanation for the increased risk of nOA lung cancers among occupationally exposed patients observed in this study. However, this finding should encourage further experimental investigations to clarify the underlying mechanisms. In addition, the poorer prognosis associated with nOA lung cancers underscores the importance of reducing occupational exposures as a key component of cancer prevention.

As outlined in Section 2 and Section 3, KRAS mutations were not systematically tested in all patients at ICS Maugeri, which may account for the difference in mutational profiles observed between the two centers. Indeed, the frequency of KRAS mutations differed significantly between the two cohorts (Table 1). This discrepancy reflects variations in diagnostic protocols: while ICS Maugeri did not routinely screen for KRAS mutations, CC-HRH systematically tested all adenocarcinoma cases. The clinical relevance of KRAS mutations—particularly the G12C variant—has primarily been recognized for its prognostic value. Accordingly, KRAS mutations were considered from a clinical rather than a statistical perspective, and patients harboring KRAS alterations alone were not classified as “oncogene-addicted”, given the established association between KRAS mutations and environmental and/or occupational exposures.

Only recently have targeted therapies, such as sotorasib and adagrasib, become available for KRAS-mutated LC, typically as second-line treatment options [18]. For this reason, KRAS-driven LCs were grouped together with other nOA tumors and analyzed accordingly.

It is also noteworthy that no significant differences in lung cancer histological subtypes were detected between patients with and without occupational exposure to carcinogens. Consistently with previous findings from the same Italian region [28], our results confirm that LC histology did not differ according to occupational exposure status (Table S1).

Interestingly, prior studies have reported that actionable mutations are more frequent among Asian populations, particularly in nonsmokers and women [29,30]. This observation aligns with the present findings: mutations associated with oncogene addiction were more common in the NE group (54.6%) compared with the LE (44.4%) and HE (22.9%) groups (*p* = 0.006). A similar distribution was observed in relation to smoking habits (*p* = 0.025).

### 4.2. Occupational Exposures

The prevalence of HE in our cohort was 23.6%, a finding broadly consistent with previous studies conducted in the Lombardy region. For instance, the EAGLE study (2012) reported that among LC cases, the prevalence of exposure to asbestos, silica, nickel–chromium, polycyclic aromatic hydrocarbons, and diesel exhausts ranged between 41.1% and 24.1% [28]. Similarly, a more recent Italian study involving 453 lung cancer patients identified occupational exposures in approximately 24.5% of cases [31]. In the present study, occupational exposure was assessed through a validated questionnaire and the ReNalOcCaM methodology [22]. We believe that the rigorous application of INAIL’s standardized approach by trained researchers provided sufficiently robust data to classify exposure levels. Detailed information on patients’ estimated exposure to specific lung carcinogens, by occupational sector and job tasks, is reported in Tables S10 and S11 (Supplementary Materials).

Although demographic, clinical, histological, and mutational features were comparable between the two study groups, occupational exposures were more frequent among ICS Maugeri patients. This difference may be explained by the fact that the ICS Maugeri population was slightly older at diagnosis, was younger at first employment, and more frequently began working before 1970—both in the overall cohort and within the adenocarcinoma subgroup. These characteristics likely reflect an increased probability or intensity of exposure during earlier decades, when regulatory measures were less stringent. Indeed, nearly 50% of HE patients in this study entered the workforce before 1970, and almost all before 1980. Furthermore, differences in HE prevalence between the two centers may also reflect socioeconomic disparities between their respective catchment areas.

In analyses restricted to female subjects, the risk of presenting with a non-oncogene-addicted (nOA) phenotype appeared higher in the low-exposure group. This may reflect greater challenges in reconstructing occupational exposure histories among women, possibly due to shorter employment durations or exposures occurring remotely earlier in life. A similar, though not statistically significant, trend was observed among patients diagnosed at ≤60 years of age (Table S8, Supplementary Materials).

It must also be considered that some individuals in this study may have experienced multiple exposures to different carcinogens, either simultaneously during the same employment period or sequentially across different jobs. Such combined exposures may have further increased risk [25], although the precise extent of this effect cannot be determined with the available data.

Furthermore, a recent French study reported that patients undergoing surgical resection for non-small-cell lung cancer who had a history of occupational exposure experienced more complex surgical courses and worse postoperative outcomes compared with nonexposed patients [32]. These findings are consistent with our observations and underscore the multifaceted clinical implications of occupational exposure.

Asbestos exposure was also considered a relevant risk factor for lung cancer in our cohort. Although extraction, commercialization, and use of asbestos were officially banned in Italy in 1992 [24], its use was widespread in the 1970s and 1980s, with per capita consumption ranging from 2 to 5 tons per 100,000 inhabitants [33]. Residual exposures after the ban cannot be excluded, particularly during maintenance of materials installed before 1992, as frequently encountered in the construction sector. Importantly, asbestos continues to be used in several countries, including Russia, China, and India [34].

### 4.3. Smoking Habits

The causal role of smoking in lung cancer has been extensively documented [35] and further corroborated by recent epidemiological studies [36]. For this reason, to disentangle the contribution of occupational exposures to the observed increased probability of developing nOA LC, additional models were applied adjusting for smoking history. Notably, when adjusting for sex, age at diagnosis, and smoking status (never, former, or current smoker at diagnosis), the elevated ORs of nOA LC among patients classified as HE to workplace carcinogens remained statistically significant. In Model 3, where smoking exposure was expressed in pack-years, a slight but non-significant increase in the OR persisted. It should be noted that the confidence intervals of ORs for the nOA phenotype in HE individuals were wide (Table 2 and Figure 1), which may partially account for these results.

Sensitivity analyses stratified by smoking status were consistent with the primary findings, showing a stronger association in current smokers and in those with heavy smoking histories (Table S9, Supplementary Materials).

Although both smoking and occupational exposure exhibited independent effects on the risk of nOA LC, interaction terms between smoking status and exposure level were not statistically significant, suggesting no evidence of effect modification in this sample. However, potential attenuation of combined effects was observed, warranting further investigation in larger cohorts (Table S6, Supplementary Materials).

### 4.4. Timing of Exposure

Our findings suggest that the timing of occupational exposure in HE patients may influence the mutational landscape of lung cancer and support the hypothesis that the temporal dimension of exposure plays a role in LC development.

As expected, all models used in this study indicated that exposure at the time of diagnosis was not associated with increased LC risk. In contrast, occupational exposures occurring 5–10 years before diagnosis or 5–10 years before estimated disease onset (as defined in Section 2) were associated with a modest, though not statistically significant, increase in risk. While this effect is small and difficult to quantify given the limited sample size, it is consistent with previous reports: a study on diesel exhaust found an especially elevated risk when exposure occurred 10–19 years before death [37], and other evidence suggests that, for comparable cigarette smoking histories, occupational exposures occurring ~20 years prior to interviews are more relevant than exposures during other time windows [38].

It is inherently impossible to determine the precise latency period of each individual lung cancer case. “True latency” is defined as the interval between the initial malignant transformation of the first neoplastic cell destined to form the tumor and the date of clinical diagnosis. Several linear models of LC development have been proposed [39], but their accuracy is limited, partly because biological evidence suggests that tumor progression more likely follows a Gompertzian growth curve [39,40]. For this reason, we adopted in the present study the Weibull model proposed by Nadler and Zurbenko [19,20] to estimate true latency. While this remains a theoretical approximation, we acknowledge the substantial variability across individual cases.

Two important issues warrant further dedicated investigation when evaluating the relationship between occupational exposure and lung cancer risk, as well as the resulting phenotypic differences, particularly the prevalence of nOA profiles. The first concerns the role of simultaneous exposures to multiple carcinogens. In a recent study by Olsson et al., synergistic effects were reported: an increased risk of lung adenocarcinoma in men exposed to both chromium(VI) and silica, and a higher risk of small-cell lung cancer in women with combined exposures to silica and asbestos, PAHs, or chromium(VI) [25].

The second issue relates to health inequalities in the burden of lung cancer attributable to occupational carcinogens. As highlighted by the Global Burden of Disease Study 2019, disparities remain a critical concern worldwide [41]. Collecting data from countries where lung cancer prevalence remains particularly high could provide valuable insights into the role of occupational and environmental exposures in shaping distinct LC phenotypes.

### 4.5. Limitations of the Study

Although the calculated sample size was adequate, the wide variability in the prevalence of major occupational carcinogen exposures limited the statistical power of some analyses. We believe that a larger study population would have allowed more conclusive results. Nevertheless, the findings remain promising, particularly when considering the entire cohort and those with the most prolonged and intense exposures. The relatively small sample size also prevented separate analyses by productive sector or by specific carcinogens. A quantitative or semi-quantitative assessment of exposure—such as through job–exposure matrices—was not feasible, as no data on exposure intensity were available for the included subjects. We acknowledge this as a major limitation, as it precluded more precise attribution of risk to specific work activities or carcinogenic agents. Still, this study should be regarded as exploratory, highlighting the importance of reconstructing occupational exposures as part of the initial appraisal of clinical complexity. This effort should complement, rather than replace, standard diagnostic investigations and medical history taking.

The potential biases inherent to retrospective exposure reconstruction are well known [42,43] and were taken into account. Identification of a single carcinogenic agent is often uncertain, as patients may lack awareness of workplace hazards and face difficulties recalling the circumstances, substances, or agents involved—particularly when exposures occurred many years earlier. Sensitivity analyses (Tables S7 and S8, Supplementary Materials) further suggest that reconstructing occupational exposure is especially challenging among women and younger patients, plausibly due to reduced familiarity with production processes, shorter employment histories, or lower-intensity exposures that may be under-recognized. Moreover, in several industrial sectors and job tasks, simultaneous exposure to multiple carcinogens is common, making it impossible to isolate individual agents. Consequently, retrospective exposure assessment and estimation of exposure duration relied on patient recall, supported by validated questionnaires [22]. Given the long latency of occupational cancers (often decades) and variability in exposure intensity and work environments, this qualitative approach—classifying each worker into one of three categories (NE, LE, HE)—was the only feasible method. However, residual misclassification of exposure cannot be excluded.

## 5. Conclusions

In the analyzed cohort, occupational exposure to recognized lung carcinogens was associated with a higher likelihood of non-oncogene-addicted (nOA) adenocarcinomas compared with no exposure. This association persisted after adjustment for age, sex, and smoking status, although it was attenuated when smoking intensity (pack-years) was modeled, underscoring the intertwined contributions of tobacco use and occupational factors. No differences were observed in histological subtype distribution, whereas sensitivity analyses highlighted greater challenges in reconstructing exposures among women and younger patients, consistent with potential differential misclassification. Collectively, these findings emphasize the importance of incorporating a standardized occupational exposure assessment—using structured questionnaires and task-specific probes—into the baseline diagnostic work-up of lung cancer, complementing molecular profiling and routine anamnesis.

Given the exploratory design, modest sample size, and qualitative exposure assessment, causal inference cannot be established, and residual confounding cannot be excluded. To enhance the robustness of future findings, multicenter prospective studies should harmonize exposure metrics (e.g., job–exposure matrices, task-specific modules, and, where feasible, exposure biomarkers), rigorously account for smoking intensity and timing, and evaluate outcomes across molecularly defined subgroups. Beyond clinical implications, systematic appraisal of occupational histories may also facilitate case recognition for compensation and strengthen epidemiological surveillance and preventive strategies in thoracic oncology.

## Figures and Tables

**Table 1:** Demographics, smoking habits, and occupational exposure of enrolled patients, by center, Pavia–Milan (Italy), 2022–2023.

	ICS Maugeri	CC-HRH	All	*p*
**Sex**				
Male	58 (58.0%)	52 (52.5%)	110 (55.3%)	
Female	42 (42.0%)	47 (47.5%)	89 (44.7%)	0.437
**Age at enrollment (years)**				
40–49	3 (3.0%)	4 (4.0%)	7 (3.5%)	
50–59	15 (15.0%)	19 (19.2%)	34 (17.1%)	
60–69	29 (29.0%)	37 (37.4%)	66 (33.2%)	
70–79	40 (40.0%)	30 (30.3%)	70 (35.2%)	
80+	13 (13.0%)	9 (9.1%)	22 (11.1%)	0.443
**Smoking habits**				
Never	12 (12.0%)	7 (7.1%)	19 (9.5%)	
Former at diagnosis				
*0–9 years before diagnosis*	19 (19.0%)	11 (11.1%)	30 (15.1%)	
*10–19 years before diagnosis*	11 (11.0%)	13 (13.1%)	24 (12.1%)	
*20+ years before diagnosis*	24 (24.0%)	17 (17.2%)	41 (20.6%)	
Current at diagnosis	34 (34.0%)	51 (51.5%)	85 (42.7%)	0.084
Pack-years (median, IQR)	40.0 (20.8–60.8)	46.0 (27.0–58.0)	42.8 (22.0–60.0)	0.508
**Occupational exposure** ^a^				
Not exposed	49 (49.0%)	69 (69.7%)	118 (59.3%)	
Low exposure	20 (20.0%)	14 (14.1%)	34 (17.1%)	
High exposure	31 (31.0%)	16 (16.2%)	47 (23.6%)	0.010

^a^ Low exposure corresponds to patients who had at least a work period with possible exposure to lung carcinogens, although its duration was briefer than the sum of other work periods without carcinogen exposure; high exposure corresponds to patients who had their longest working period characterized by probable exposure to lung carcinogens.

**Table 2:** Lung cancer characteristics included histological and gene mutation features. Pavia–Milan (Italy), 2022–2023.

	ICS Maugeri	CC-HRH	All	*p*
**Age at diagnosis (years)**				
40–49	1 (1.0%)	11 (11.1%)	12 (6.0%)	
50–59	22 (22.0%)	16 (16.2%)	38 (19.1%)	
60–69	31 (31.0%)	44 (44.4%)	75 (37.7%)	
70–79	38 (38.0%)	23 (23.2%)	61 (30.7%)	
80+	8 (8.0%)	5 (5.1%)	13 (6.5%)	0.003
**Histological type**				
Adenocarcinoma	78 (78.0%)	72 (73.4%)	150 (75.8%)	
Squamous	13 (13.0%)	13 (13.3%)	26 (13.1%)	
Small cell	9 (9.0%)	10 (10.2%)	19 (9.6%)	
Others	0 -	3 (3.1%)	3 (1.5%)	0.351
**Gene mutations ^a^**				
EGFR	5 (6.4%)	9 (12.5%)	14 (9.3%)	0.200
ALK	5 (5.4%)	1 (1.4%)	6 (4.0%)	0.117
ROS1	2 (2.6%)	1 (1.4%)	3 (2.0%)	0.608
BRAF	4 (5.1%)	5 (6.9%)	9 (6.0%)	0.640
KRAS	8 (10.3%)	27 (37.5%)	35 (23.3%)	<0.001
RET	1 (1.3%)	0 -	1 (0.7%)	0.335
MET	0 -	3 (4.2%)	3 (2.0%)	0.069
HER2	0 -	3 (4.2%)	3 (2.0%)	0.359

^a^ Only adenocarcinoma patients: ICS Maugeri, *n* = 78; CC-HRH, *n* = 72; total *n* = 150.

**Table 3:** Odds ratio and 95% confidence interval of non-oncogene-addicted phenotype by exposure to occupational lung carcinogens, in adenocarcinoma cases of ICS Maugeri, of Cancer Center of Humanitas Research Hospital (CC-HRH), and in the entire population.

Center	*n*	Model 1	*p*	Model 2	*p*	Model 3	*p*
		OR (95%IC)		OR (95%IC)		OR (95%IC)	
**ICS Maugeri**							
Never Exposed	37	1 (ref.)	-	1 (ref.)	-	1 (ref.)	-
Low Exposure	17	1.46 (0.42–5.03)	0.548	1.25 (0.31–5.08)	0.751	1.79 (0.48–6.67)	0.383
High Exposure	24	6.70 (1.36–32.92)	0.019	5.94 (1.05–33.51)	0.044	2.97 (0.45–19.29)	0.255
			*t*: 0.02		*t*: 0.04		*t*: 0.26
**CC-HRH**							
Never Exposed	51	1 (ref.)	-	1 (ref.)	-	1 (ref.)	-
Low Exposed	10	0.85 (0.20–3.73)	0.873	0.69 (0.15–3.19)	0.630	0.80 (0.17–3.77)	0.781
High Exposed	11	1.67 (0.44–6.25)	0.449	1.08 (0.26–4.51)	0.914	1.08 (0.25–4.63)	0.914
			*t*: 0.45		*t*: 0.91		*t*: 0.91
**Total**							
Never Exposed	88	1 (ref.)	-	1 (ref.)	-	1 (ref.)	-
Low Exposed	27	1.50 (0.63–3.57)	0.360	1.50 (0.60–3.75)	0.383	1.71 (0.68–4.30)	0.251
High Exposed	35	4.05 (1.66–9.90)	0.002	3.07 (1.16–8.11)	0.023	2.08 (0.74–5.82)	0.162
			*t*: <0.01		*t*: 0.02		*t*: 0.16

Patients are classified as never exposed (NE), low exposed (LE), and high exposed (HE) to occupational carcinogens as defined in the text. In Model 1, the data are unadjusted; in Model 2, the data are adjusted for sex, age at diagnosis, and smoking habits (never, former, and current smokers at diagnosis); in Model 3, the data are adjusted for sex, age at diagnosis, and smoking habits in cigarette pack-years.

## Data Availability

The original contributions presented in this study are included in the article and Supplementary Materials. Further inquiries can be directed to the corresponding author.

## Supplementary Materials

The following supporting information can be downloaded at https://www.mdpi.com/article/10.3390/cancers17182997/s1. Table S1. Lung cancer histotypes by lung carcinogen exposure, Pavia–Milan (Italy), 2022–2023. Table S2. Possible occupational exposure to lung carcinogens at specific time points, by center, Pavia–Milano, Italy, 2022–2023. Table S3. Risk of non-oncogene-addicted phenotype of LC by exposure to occupational lung carcinogens at different time points in patients with adenocarcinoma diagnosis, Pavia–Milan (Italy), 2022–2023. Table S4. Results of ’model diagnostic, Pavia–Milan (Italy), 2022–2023. Table S5. Test for collinearity: values of variance inflation factor (VIF), by center and total. Pavia–Milan (Italy), 2022–2023. Table S6. Estimates for interaction (smoke x exposure). Pavia–Milan (Italy), 2022–2023. Table S7. Odds ratio (OR) of non-oncogene-addicted (nOA) phenotype by exposure to occupational lung carcinogens, among patients with adenocarcinoma, by sex. Pavia–Milan (Italy), 2022–2023. Table S8. Odds ratio (OR) of non-oncogene-addicted (nOA) phenotype by exposure to occupational lung carcinogens, among patients with adenocarcinoma, by age class (<60, 60–69, 70+) at diagnosis. Pavia–Milan (Italy), 2022–2023. Table S9. Odds ratio (OR) of non-oncogene-addicted (nOA) phenotype by exposure to occupational lung carcinogens, among patients with adenocarcinoma, by smoke habits (never, former, present, or under or above median of 42.75 py). Pavia–Milan (Italy), 2022–2023. Table S10. Occupational sectors, job tasks, and relative exposures/epidemiological data retrieved from the literature. Table S11. Job tasks and duration, and main exposure of workers classified as high exposed. Pavia–Milan (Italy), 2022–2023.

## Abbreviations

The following abbreviations are used in this manuscript:

LC	Lung cancer
SCLC	Small-cell lung cancer
NSCLC	Non-small-cell lung cancer
NGS	Next-generation sequencing
ISIC	International Standard Industrial Classification of All Economic Activities
INAIL	Istituto Nazionale per l’Assicurazione per gli Infortuni sul Lavoro
IARC	International Agency for Research on Cancer
EGFR	Epidermal Growth Factor Receptor
ALK	Anaplastic Lymphoma Kinase
ROS1	ROS proto-oncogene 1, receptor tyrosine kinase
BRAF	B-Raf proto-oncogene, serine/threonine kinase
KRAS	Kirsten rat sarcoma viral oncogene
OA	Oncogene-addicted
nOA	Non-oncogene-addicted
