# Supplementary material for: Exposure to Occupational Carcinogens and Non-Oncogene Addicted Phenotype in Lung Cancer: Results from a Real-Life Observational Study
Source: Cancers (Basel). 2025 Sep 13;17(18):2997. doi: 10.3390/cancers17182997 (PMC12468263; doi:10.3390/cancers17182997)
Supplement: Supplementary file 1 [file cancers-17-02997-s001.zip › Table S1.pdf]

Table S1. Lung cancer histotypes by lung carcinogen exposure, Pavia-Milan (Italy), 2022-2023.

	NE	LE	HE	*p*
**Histological type**				
Adenocarcinoma	88 (75.2%)	27 (79.4%)	35 (74.5%)	
Squamous	15 (12.8%)	5 (14.7%)	6 (12.8%)	
Small cell	13 (11.1%)	1 (2.9%)	5 (10.6%)	
Others	2 (1.7%)	1 (2.9%)	1 (2.1%)	0.813

HE: higher exposed; LE: lower exposed; LE non-exposed
