# Supplementary material for: Exposure to Occupational Carcinogens and Non-Oncogene Addicted Phenotype in Lung Cancer: Results from a Real-Life Observational Study
Source: Cancers (Basel). 2025 Sep 13;17(18):2997. doi: 10.3390/cancers17182997 (PMC12468263; doi:10.3390/cancers17182997)
Supplement: Supplementary file 1 [file cancers-17-02997-s001.zip › Table S2.pdf]

Table S2. Possible occupational exposure to lung carcinogens at specific time points, by center, Pavia-Milano, Italy, 2022-2023.

Occupational exposure	ICS Maugeri (Yes/No)	IC Humanitas (Yes/no)	Total (Yes/no)	*p*
At the LC onset [a]	21/79	19/80	40/159	0.750
5 years before onset	21/79	19/80	40/159	0.750
10 years before onset	23/77	18/81	41/158	0.401
At time of LC diagnosis	4/96	6/93	10/189	0.506
5 years before diagnosis	10/90	9/90	19/180	0.827
10 years before diagnosis	13/87	12/87	25/174	0.852

[a]: Onset of LC was defined as the point in time 13.6 years before clinical diagnosis, according to Nadler and Zurbenko [20].
