# Supplementary material for: Exposure to Occupational Carcinogens and Non-Oncogene Addicted Phenotype in Lung Cancer: Results from a Real-Life Observational Study
Source: Cancers (Basel). 2025 Sep 13;17(18):2997. doi: 10.3390/cancers17182997 (PMC12468263; doi:10.3390/cancers17182997)
Supplement: Supplementary file 1 [file cancers-17-02997-s001.zip › Table S3.pdf]

Table S3. Risk of non-oncogene addicted phenotype of LC by exposure to occupational lung carcinogens at different time-points in patients with adenocarcinoma diagnosis, Pavia-Milan (Italy), 2022-2023.

		Model 1		Model 2		Model 3	
	n	OR (95%IC)	p	OR (95%IC)	p	OR (95%IC)	p
Not exposed[a]	121	1 (ref.)	-	1 (ref.)	-	1 (ref.)	-
Exposed at onset	29	1.75 (0.75-4.07)	0.194	1.36 (0.55-3.35)	0.507	0.83 (0.31-2.26)	0.722
Not exposed[b]	121	1 (ref.)	-	1 (ref.)	-	1 (ref.)	-
Exposed 5 years before onset	29	1.75 (0.75-4.07)	0.194	1.36 (0.55-3.35)	0.507	0.83 (0.31-2.26)	0.722
Not exposed[c]	120	1 (ref.)	-	1 (ref.)	-	1 (ref.)	-
Exposed 10 years before onset	30	1.87 (0.80-4.33)	0.143	1.42 (0.58-3.49)	0.442	0.85 (0.31-2.32)	0.755
Not exposed[d]	141	1 (ref.)	-	1 (ref.)	-	1 (ref.)	-
Exposed at diagnosis	9	0.64 (0.17-2.51)	0.528	0.71 (0.17-3.00)	0.642	0.61 (0.13-2.71)	0.512
Not exposed[e]	134	1 (ref.)	-	1 (ref.)	-	1 (ref.)	-
Exposed 5 years before diagnosis	16	1.44 (0.49-4.17)	0.507	1.47 (0.47-4.57)	0.511	0.96 (0.28-3.29)	0.946
Not exposed[f]	130	1 (ref.)	-	1 (ref.)	-	1 (ref.)	-
Exposed 10 years before diagnosis	20	2.13 (0.77-5.88)	0.145	2.07 (0.70-6.15)	0.187	1.45 (0.46-4.56)	0.530

Model 1: Unadjusted; Model 2: Adjusted for gender, age at diagnosis and smoke habits (never, former and current smokers at diagnosis); Model 3: Adjusted for gender, age at diagnosis and pack-years.

t: test for linear trend

[a]: Never exposed + not exposed at onset; [b]: Never exposed + not exposed 5 years before onset; [c]: Never exposed + not exposed 10 years before onset; [d]: Never exposed + not exposed at diagnosis.; [e]: Never exposed + not exposed 5 years before diagnosis.; [f]: Never exposed + not exposed 10 years before diagnosis.
