# Supplementary material for: Exposure to Occupational Carcinogens and Non-Oncogene Addicted Phenotype in Lung Cancer: Results from a Real-Life Observational Study
Source: Cancers (Basel). 2025 Sep 13;17(18):2997. doi: 10.3390/cancers17182997 (PMC12468263; doi:10.3390/cancers17182997)
Supplement: Supplementary file 1 [file cancers-17-02997-s001.zip › Table S4.pdf]

Table S4. Results of model diagnostic, Pavia-Milan (Italy), 2022-2023.

	Model 1		Model 2		Model 3	
**Center**	**OR (95%IC)**	**p**	**OR (95%IC)**	**p**	**OR (95%IC)**	**p**
**ICS Maugeri**						
Never Exposed	1 (ref.)	-	1 (ref.)	-	1 (ref.)	-
Low Exposure	1.46 (0.42-5.03)	0.548	1.25 (0.31-5.08)	0.751	1.79 (0.48-6.67)	0.383
High Exposure	6.70 (1.36-32.92)	0.019	5.94 (1.05-33.51)	0.044	2.97 (0.45-19.29)	0.255
Goodness-of-fit		*<0.05*		*0.279*		*0.077*
Residuals stand. ±2 (n/total)		*96/150*		*76/150*		*75/150*
Residuals stand. ±3 (n/total)		*96/150*		*76/150*		*75/150*
**CC-HRH**						
Never Exposed	1 (ref.)	-	1 (ref.)	-	1 (ref.)	-
Low Exposed	0.85 (0.20-3.73)	0.873	0.69 (0.15-3.19)	0.630	0.80 (0.17-3.77)	0.781
High Exposed	1.67 (0.44-6.25)	0.449	1.08 (0.26-4.51)	0.914	1.08 (0.25-4.63)	0.914
Goodness-of-fit		*<0.05*		*0.627*		*0.481*
Residuals stand. ±2 (n/total)		*139/150*		*79/150*		*79/150*
Residuals stand. ±3 (n/total)		*139/150*		*78/150*		*78/150*
**Total**						
Never exposed	1 (ref.)	-	1 (ref.)	-	1 (ref.)	-
Low Exposed	1.50 (0.63-3.57)	0.360	1.50 (0.60-3.75)	0.383	1.71 (0.68-4.30)	0.251
High Exposed	4.05 (1.66-9.90)	0.002	3.07 (1.16-8.11)	0.023	2.08 (0.74-5.82)	0.162
Goodness-of-fit		*<0.05*		*0.117*		*0.294*
Residuals stand. ±2 (n/total)		*88/150*		*2/150*		*1/150*
Residuals stand. ±3 (n/total)		*62/150*		*0/150*		*1/150*

Model 1: Unadjusted; Model 2: Adjusted for sex, age at diagnosis and smoke habits (never, former and current smokers at diagnosis); Model 3: Adjusted for sex, age at diagnosis and smoke habits as pack-years. Godness-of-fit calculated with the test di Hosmer-Lemeshow.
