# Supplementary material for: Exposure to Occupational Carcinogens and Non-Oncogene Addicted Phenotype in Lung Cancer: Results from a Real-Life Observational Study
Source: Cancers (Basel). 2025 Sep 13;17(18):2997. doi: 10.3390/cancers17182997 (PMC12468263; doi:10.3390/cancers17182997)
Supplement: Supplementary file 1 [file cancers-17-02997-s001.zip › Table S5.pdf]

Table S5. Test for collinearity: values of variance inflation factor (VIF), by center and total. Pavia-Milan (Italy), 2022-2023.

Center	VIF-Model2	1/VIF-Model 2	VIF-Model3	1/VIF-Model 3
**ICS Maugeri**				
Sex	1.50	0.67	1.37	0.73
Smoke	1.33	0.75	1.62	0.62
Age at diagnosis	1.18	0.85	1.15	0.87
Low Exposure	1.38	0.73	1.24	0.81
High Exposure	1.47	0.68	1.94	0.51
**CC-HRH**				
Sex	1.23	0.81	1.22	0.82
Smoke	1.20	0.83	1.11	0.90
Age at diagnosis	1.21	0.83	1.11	0.90
Low Exposure	1.10	0.91	1.10	0.91
High Exposure	1.19	0.84	1.18	0.85
**Total**				
Sex	1.35	0.74	1.28	0.78
Smoke	1.20	0.83	1.37	0.73
Age at diagnosis	1.17	0.85	1.12	0.89
Low Exposure	1.15	0.87	1.14	0.88
High Exposure	1.31	0.76	1.52	0.66

Model 2: sex, age at diagnosis and smoke habits (never, former and current smokers at diagnosis); Model 3: sex, age at diagnosis and smoke habits as pack-years.
