# Supplementary material for: Exposure to Occupational Carcinogens and Non-Oncogene Addicted Phenotype in Lung Cancer: Results from a Real-Life Observational Study
Source: Cancers (Basel). 2025 Sep 13;17(18):2997. doi: 10.3390/cancers17182997 (PMC12468263; doi:10.3390/cancers17182997)
Supplement: Supplementary file 1 [file cancers-17-02997-s001.zip › Table S6.pdf]

Table S6. Estimates for interaction (smoke x exposure). Pavia-Milan (Italy), 2022-2023.

Non-Oncogene addiction	OR	95% confidence interval	p
**Smoke (py)**	1.02	1.00-1.04	0.026
**Exposure**			
High	4.04	0.81-20.14	0.089
Low	2.42	0.42-14.04	0.324
**Exposure x Smoke (py)**			
High	0.97	0.93-1.01	0.191
Low	0.99	0.97-1.03	0.943
**Smoke (Never, Former, Current)**			
Current	7.65	1.87-31.33	0.005
Former	4.44	1.09-18.18	0.038
**Exposure**			
High	10.00	0.74-135.33	0.083
Low	3.33	0.16-70.91	0.440
**Exposure x Smoke**			
High x Current	0.09	0.01-2.06	0.130
High x Former	0.13	0.01-2.04	0.144
Low x Current	1.37	0.04-42.46	0.856
Low x Former	0.77	0.03-20.03	0.872

*Smoke habits tested both as continuous variable (pack-years, py) and as categorical variable (never, former, present smoker).
