# Supplementary material for: Exposure to Occupational Carcinogens and Non-Oncogene Addicted Phenotype in Lung Cancer: Results from a Real-Life Observational Study
Source: Cancers (Basel). 2025 Sep 13;17(18):2997. doi: 10.3390/cancers17182997 (PMC12468263; doi:10.3390/cancers17182997)
Supplement: Supplementary file 1 [file cancers-17-02997-s001.zip › Table S7.pdf]

Table S7. Odds Ratio (OR) of non-oncogene addicted (nOA) phenotype by exposure to occupational lung carcinogens, among patients with adenocarcinoma, by sex. Pavia-Milan (Italy), 2022-2023.

	Model 1		Model 2a		Model 3a	
**Sex**	**OR (95%IC)**	**p**	**OR (95%IC)**	**p**	**OR (95%IC)**	**p**
**Male**						
Never Exposed	1 (ref.)	-	1 (ref.)	-	1 (ref.)	-
Low Exposure	0.70 (0.22-2.26)	0.552	0.86 (0.26-2.91)	0.814	0.80 (0.24-2.69)	0.730
High Exposure	3.29 (1.07-10.07)	0.037	2.99 (0.96-9.33)	0.059	2.72 (0.79-9.43)	0.114
Goodness-of-fit		*<0.05*		*0.500*		*0.316*
**Female**						
Never Exposed	1 (ref.)	-	1 (ref.)	-	1 (ref.)	-
Low Exposed	3.67 (0.85-15.77)	0.081	3.57 (0.79-16.14)	0.097	6.02 (1.25-28.99)	0.025
High Exposed	1.57 (0.20-12.02)	0.663	1.77 (0.22-14.20)	0.590	0.65 (0.05-9.14)	0.752
Goodness-of-fit		*<0.05*		*0.208*		*0.336*

Model 1: Unadjusted; Model 2a: Adjusted for age at diagnosis and smoke habits (never, former and current smokers at diagnosis); Model 3a: Adjusted for age at diagnosis and smoke habits as pack-years. Godness-of-fit calculated with the test di Hosmer-Lemeshow.
