# Supplementary material for: Exposure to Occupational Carcinogens and Non-Oncogene Addicted Phenotype in Lung Cancer: Results from a Real-Life Observational Study
Source: Cancers (Basel). 2025 Sep 13;17(18):2997. doi: 10.3390/cancers17182997 (PMC12468263; doi:10.3390/cancers17182997)
Supplement: Supplementary file 1 [file cancers-17-02997-s001.zip › Table S8.pdf]

Table S8. Odds Ratio (OR) of non-oncogene addicted (nOA) phenotype by exposure to occupational lung carcinogens, among patients with adenocarcinoma, by age class (<60, 60-69, 70+) at diagnosis. Pavia-Milan (Italy), 2022-2023.

	**Model 1**		**Model 2b**		**Model 3b**	
**Age at diagnosis**	**OR (95%IC)**	**p**	**OR (95%IC)**	**p**	**OR (95%IC)**	**p**
**<60**						
Never Exposed	1 (ref.)	-	1 (ref.)	-	1 (ref.)	-
Low Exposure	3.60 (0.74-17.60)	0.114	4.82 (0.78-29.73)	0.090	5.13 (0.89-29.66)	0.068
High Exposure	2.70 (0.38-18.96)	0.318	2.26 (0.27-18.68)	0.451	1.60 (0.18-14.51)	0.678
Goodness-of-fit		*<0.05*		*0.219*		*0.104*
**60-69**						
Never Exposed	1 (ref.)	-	1 (ref.)	-	1 (ref.)	-
Low Exposed	1.59 (0.45-5.57)	0.468	1.25 (0.28-5.53)	0.767	1.17 (0.29-4.77)	0.829
High Exposed	9.09 (1.68-49.12)	0.010	5.15 (0.84-31.58)	0.077	4.43 (0.68-28.98)	0.121
Goodness-of-fit		*<0.05*		*0.119*		*0.394*
**70+**						
Never Exposed	1 (ref.)	-	1 (ref.)	-	1 (ref.)	-
Low Exposed	1.05 (0.08-13.08)	0.968	1.09 (0.08-14.80)	0.946	1.23 (0.10-15.76)	0.873
High Exposed	1.84 (0.48-7.10)	0.375	1.86 (0.42-8.34)	0.414	1.33 (0.27-6.65)	0.722
Goodness-of-fit		*<0.05*		*0.627*		*0.349*

Model 1: Unadjusted; Model 2b: Adjusted for sex and smoke habits (never, former and current smokers at diagnosis); Model 3b: Adjusted for sex and smoke habits as pack-years. Godness-of-fit calculated with the test di Hosmer-Lemeshow.
