# Supplementary material for: Exposure to Occupational Carcinogens and Non-Oncogene Addicted Phenotype in Lung Cancer: Results from a Real-Life Observational Study
Source: Cancers (Basel). 2025 Sep 13;17(18):2997. doi: 10.3390/cancers17182997 (PMC12468263; doi:10.3390/cancers17182997)
Supplement: Supplementary file 1 [file cancers-17-02997-s001.zip › Table S9.pdf]

Table S9. Odds Ratio (OR) of non-oncogene addicted (nOA) phenotype by exposure to occupational lung carcinogens, among patients with adenocarcinoma, by smoke habits (never, former, present, or under of above media of 42.75 py). Pavia-Milan (Italy), 2022-2023.

	**Model 1**		**Model 2c**	
**Smoke habits**	**OR (95%IC)**	**p**	**OR (95%IC)**	**p**
**Never***				
Never Exposed	1 (ref.)	-	1 (ref.)	-
Low Exposure	10.00 (0.58-171.2)	0.112	-	-
High Exposure	-	-	-	-
Goodness-of-fit		*<0.05*		-
**Former**				
Never Exposed	1 (ref.)	-	1 (ref.)	-
Low Exposed	1.35 (0.46-3.97)	0.468	1.40 (0.47-4.21)	0.550
High Exposed	2.65 (0.79-8.93)	0.010	2.14 (0.57-8.11)	0.263
Goodness-of-fit		*<0.05*		*0.627*
**Present**				
Never Exposed	1 (ref.)	-	1 (ref.)	-
Low Exposed	0.89 (0.11-7.06)	0.916	0.88 (0.10-7.86)	0.913
High Exposed	6.71 (1.33-33.71)	0.021	5.44 (0.95-31.1)	0.057
Goodness-of-fit		*<0.05*		*0.772*
**Pack-years <42.75 (median)**				
Never Exposed	1 (ref.)	-	1 (ref.)	-
Low Exposure	2.60 (0.90-7.50)	0.076	2.68 (0.88-8.18)	0.082
High Exposure	2.17 (0.52-9.08)	0.288	1.50 (0.32-7.11)	0.608
Goodness-of-fit		*<0.05*		*0.501*
**Pack-years >=42.75 (median)**				
Never Exposed	1 (ref.)	-	1 (ref.)	-
Low Exposed	0.54 (0.10-2.75)	0.455	0.58 (0.10-3.23)	0.533
High Exposed	3.93 (1.12-13.78)	0.033	3.93 (0.98-15.84)	0.054
Goodness-of-fit		*<0.05*		*0.362*

Model 1: Unadjusted; Model 2c: Adjusted for sex and age at diagnosis. Godness-of-fit calculated with the test di Hosmer-Lemeshow.
*High exposure predicts failure perfectly.
