# Supplementary material for: Exposure to Occupational Carcinogens and Non-Oncogene Addicted Phenotype in Lung Cancer: Results from a Real-Life Observational Study
Source: Cancers (Basel). 2025 Sep 13;17(18):2997. doi: 10.3390/cancers17182997 (PMC12468263; doi:10.3390/cancers17182997)
Supplement: Supplementary file 1 [file cancers-17-02997-s001.zip › Table S10.pdf]

Table S10. Occupational sectors, job tasks and relative exposures/epidemiological data retrieved from literature.

ISIC Code	Description	Job tasks	Exposure	Exposure assessment	Reference	Note	Epidemiological evidence (Italy)	Reference	Note	Epidemiological evidence (Western Countries)	Reference	Note
4923	Freight transport by road	Driving and basic maintenance of trucks	•Diesel exhaust	•1-22 µg/m3 EC<br>•18.3 µg/m^3 (SD 2) EC<br>•1 µg/m^3 (SD 2.8) EC<br>•1 µg/m^3 (SD 2.3) EC	•Pronk et al. 2009[1]<br>•Boffetta et al. 2002[2]<br>•Davis et al.2007[3]13/09/2025 19:07:00	•United States<br>•Russia; Short haul drivers<br>•United States; Truck local<br>United States; Truck long haul	•RR 2.08 (90% CI 1.62-2.67)<br>•SMR 3.39 (p=0.02)<br>SMR 4.16 (p<0.01)<br>SMR 1.36 (p=0.01)	•Crosignani et al. 2006[4]<br>•Lagorio et al. 1995[5]	•Trasportation (general)<br>•Transportation (general)<br>Transportation (general)<br>Drivers	•SIR 1.13 (95% CI 1.04-1.22)	•Guo et al. 2004[6]	•Truck drivers exposed to DEE
8423	Public order and safety activities	Urban police officer with ongoing responsibilities for traffic management and direction	•Diesel exhaust	•128 µg/m^3 (±60) PMresp<br>10.9 µg/m^3 (±5.3) B	•Cattaneo et al. 2010[7]	•Italy				•OR 1.6 (95% CI 1.1-2.3)<br>•HR 1.03 (95% CI 1.00-1.06)<br>HR 1.03 (95% CI 1.00-1.07)	•Zahm et al. 1989[8]<br>•Chen et al. 2024[9]	•Policemen and firefighters<br>•NO2 esposed in Rome<br>Black Carbon exposed in Rome
432	Electrical, plumbing and other construction installation activities	General full or partial activities involving construction, finishing, maintenance, repair, demolition, and refurbishment	•Asbestos (various forms)<br>•Crystalline silica<br>•Combustion-derived soot<br>•Welding fumes	•0.13 ff/cc Asb<br>0.11 ff/cc Asb<br>•0.01–2.8 ff/cc Asb	•Paik et al. 1983[10]<br>•CONSAD 1990[13]13/09/2025 19:07:00	•United States; Electricians<br>United States; Plumbers<br>•United States; Building maintenance	• OR 2.6 (95% CI 1.0-6.4)<br>RR 1.28 (90% CI 1.17-1.41)<br>•RR 1.67 (90% CI 1.40-1.99)<br>•SMR 1.39 (95% CI 1.17-1.64)	• Richiardi et al. 2004[11]<br>•Crosignani et al. 2006[4]<br>• Scarselli et al. 2011[12]	• Plumber and pipe fitters<br>• Asbestos exposure<br>• Asbestos exposure<br>• Silica exposure			
2030	Manufacture of man-made fibres	Electrical technician in a rayon manufacturing plant	Asbestos (various form)	•0.13 ff/cc Asb	•Paik et al. 1983[10]	•United States; Electricians	•RR 1.52 (95% CI 1.00-2.25)	• Paci et al. 1987[14]	•Asbestos exposure			
2220	Manufacture of plastics products	Worker engaged in the production of plastic forniture	Plastics, resins and pigments for plastic products manufacture	•NA	-		•SMR 2.05 (p<0.05)<br>•PMR 1.37 (95% CI 1.08-1.75)	•Ietri et al. 1997[15]<br>•D'Errico et al, 2005[16]	•Rubber and plastics workers<br>•Rubber and plastics workers			
015	Growing of non-perennial crops	Driving and basic maintenance of tractors and other agricoltural machines<br>Soil preparation and tillage; subsequent operations (fertilization, sowing, etc.); harvesting and primary processing of the crop.	Diesel exhaust<br>Arsenic inorganic compounds<br>Crystalline silica<br>Combustion-derived soot	•27 µg/m^3 EC<br>27 µg/m^3 EC<br>4 µg/m^3 EC<br>4 µg/m^3 EC	•Plato et al. 2020[17]	•Sweden (1975); Farm manager<br>Sweden (1975); Farm worker<br>Sweden (2000); Farm manager<br>Sweden (2000); Farm worker	•SMR 1.59 (IC95% IC 1.12-2.38)<br>•SMR 1.26 (95%CI 1.05-1.54)	•Brianesco et al. 2014[18]<br>•Gianicolo et al. 2019[19]	•Agricoltural workers<br>•Arsenic exposed workers			
0210	Silviculture and other forestry activities	Use of chainsaw or other tools for tree felling or cutting, stump removal and root grubbing	Diesel exhaust<br>PAH	•25.9 µg/m^3 (±48.2) cPAH<br>384.2 µg/m^3 (±411.9) BTEX<br>•36.66 ppm (±16.09) CO<br>2.81 ppm (±2.18) VOCs<br>1.16 ppm (±0.26) B	•Neri et al. 2016[20]<br>•D'Antonio et al. 2024[21]	•Italy; Chainsaw use<br>•Italy; Felling (Chainsaw use)						

				20.21 ppm (±5.02) CO<br>0.6 ppm (±0.44) VOCs<br>0.6 ppm (±0.33) B<br>27.08 ppm (±1.92) CO<br>0.76 ppm (±0.578) VOCs<br>0.98 ppm (±0.28) B		Delimbing (Chainsaw use)<br><br>Bucking (Chainsaw use)						
4100	Construction of buildings	Construction workers (civil, rural, and industrial): building and demolition of structures, underground construction works, restoration, and remediation of buildings.	Asbestos (various forms)<br>Crystalline silica<br>Combustion-derived soot<br>Diesel exhaust<br>Lead chromate<br>Pitch, coal tar<br>Welding fumes	•8 $\mu g/m^3$ (SD 2.8) EC<br><br>•0.061–0.142 mg/m^3 (8h average) CS<br>0.118 mg/m^3 (8h average) CS<br><0.722 mg/m^3 (8h average) CS	•Lewné et al. 2007[22]<br><br>•INAIL 2024[24]	•Sweden; Above ground workers<br>•Italy; Building demolition<br><br>Italy; Interior renovation<br><br>Italy; Manual/mechanical cutting of walls and floors	•OR 1.57 (95% CI 1.12-2.21)<br>•OR 1.4 (95% CI 1.1-1.9)<br><br>•PMR 1.16 (95% CI 1.09-1.23)<br><br>•HR 1.27 (95%CI 1.12–1.44)	•Consonni et al. 2012[23]<br>• Richiardi et al. 2004[11]<br><br>•D'Errico et al, 2005[16]<br><br>•Massari et al. 2025[25]	•Briclayers<br>•Construction workers<br>•Construction workers<br>•Construction workers			
4930	Transport via pipeline	Fuel station operator and attendant selling gasoline and diesel	Diesel exhaust	•46 $\mu g/m^3$ EC	•Plato et al. 2020[17]	•Sweden (1950); Pump attendant	•OR 6.64 (95% CI 1.58-27.94)	•Consonni et al. 2010[26]	•Filling station attendants			
2211	Manufacture of rubber tyres and tubes	Worker involved in raw rubber processing; manufacturing of products and semi-finished goods made of or primarily composed of natural or synthetic rubber; production, regeneration, and retreading of tires	Bis(chloromethyl) ether<br>Pigments for rubber production<br>Tremolite and asbestos fiber-containg talc	•NA			•SMR 2.05 (p<0.05)<br><br>•SMR 1,67 (95% CI 1,05-2,65)<br><br>•PMR 1.37 (95% CI 1.08-1.75)	•Ietri et al. 1997[15]<br><br>•Gerosa et al. 2017[27]<br><br>•D'Errico et al, 2005[16]	•Rubber and plastics workers<br>•Rubber workers<br><br>•Rubber and plastics workers			
2750	Manufacture of domestic appliances	Worker involved in the production and assembly of household appliances	Welding fumes<br>Crystalline silica	•NA			•SMR 3.90 (p=0.05)	•Lagorio et al. 1995[5]	•Household appliances maintenance			
4210	Construction of roads and railways	Excavator machine operators and workers involved in road asphalting and surfacing	PAH<br>Diesel exhaust<br>Asbestos (various forms)<br>Crystalline silica<br>Pitch, coal tar	•135 $\mu g/m^3$ EC<br><br>20 $\mu g/m^3$ EC<br><br>•<0.01 mg/m^3 CS	•Plato et al. 2020[17]<br><br><br>•INAIL 2024[24]	•Sweden (1975); Paving and asphalt<br>Sweden (2000); Paving and asphalt<br><br>Italy (2024); Road paving/milling				•RR 1.33 (95%CI 1.20-147)<br><br>•SIR 1.13 (95% CI 1.04-1.22)	•Mundt et al. 2018[28]<br><br>•Guo et al. 2004[6]	•Bitumen-exposed workers<br>•Asphalt workers exposed to Diesel exhaust
2431	Casting of iron and steel	Metal foundry workers	Crystalline silica<br>Chromium IV and chromium compounds<br>Nichel and nichel compounds	•0.057 mg/m3 (8h average) CS	•Scarselli et al. 2011[12]	•Italy; Foundry industry	•RR 3.43 (p=0.01)<br><br>•RR 3.67 (90% IC 2.62-5.15)	•Lagorio et al. 1995[5]<br><br>•Crosignani et al. 2006[4]	•Foundry workers<br>•Iron and steel foundries			

2599	Manufacture of other fabricated metal products; metalworking service activities	Mechanical and metalworking industry workers	Welding fumes<br>Crystalline silica<br>Asbestos (various forms)<br>Chromium IV and chromium compounds<br>Nichel and nichel compounds	•0.23-3.26 mg/m3 WF<br>0.16-6.88 μg/m3 ChVI<br>•0.048 mg/m^3 (8h average) CS	•Lombardy Region 2012[29]<br>•Scarselli et al. 2011[12]	•Italy; Stainless steel welding<br>•Italy; Manufacture of basic metals	•RR 1.50 (90% IC 1.24-1.82)	•Crosignani et al. 2006[4]	•Metalworking			
1811	Printing and service activities related to printing	Workers specialized in lithography and engraving	Chromium IV and chromium compounds<br>Nichel and nichel compounds	•NA						•SIR 1.26 (95% CI 1.12–1.42)<br>•SMR 5.8 (95%CI 1.19-16.9)	•Kvam et al. 2005[30]<br>•Luce et al. 1997[31]	•Printing workers<br>•Pressmen (printing industry)
5221	Service activities incidental to land transportation	Railway staff	Diesel exhaust	•3 μg/m^3 (SD 1.5) EC<br>•6 μg/m^3 (SD -) EC	•Verma et al. 2003[32]<br>•Seshagiri et al. 2003[34]	•Canada; Hostler<br>•Canada; Non-operative crew on train				•RR 1.21 (p=0.002)	•Crump 1999[33]	•Train riders exposed to diesel exhaust
4912	Freight rail transport	Train operators and railway maintenance workers	Diesel exhaust<br>Asbestos (various forms)	•12.3 μg/m^3 (SD 1.9) EC<br>•3 μg/m^3 (SD 2.4-2.7) EC	•Boffetta et al. 2002[2]<br>•Verma et al. 2003[32]	•Russia; Shunting locomotive drivers<br>•Canada; Maintenance rolling equipment	• SMR 1.26 (95%CI 1.01-1.54)	•Tessari et al. 2004[35]	•Reparation of railway rolling stock			
3510	Electric power generation, transmission and distribution	Workers involved in the production and distribution of electric power; workers responsible for the maintenance of electric power generation facilities	Asbestos (various form)	•0.001-0.0190 ff/cc<br>•0.01-0.04ff/cc<br>•2.1 ff/cc (before 1992)	•INAIL-CONTARP 1997[36]<br>•Region of Tuscany, 1997[37]<br>•Felten et al., 2010[38]							
4520	Maintenance and repair of motor vehicles	Automotive mechanics and related motor vehicle service/repair personnel	Welding fumes<br>Diesel exhaust	•1 μg/m^3 (SD 2.3) EC<br>•1 μg/m^3 (SD 2.3) EC	•Boffetta et al. 2002[2]<br>•Groves and Cain 2002[41]	•Estonia; Bus mechanics<br>•United States; Bus mechanics				•HR 1.09 (95% CI 1.03-1.14)<br>•RR 1.16 (95% CI 1.00–1.34)<br>•SIR 1.13 (95% CI 1.04-1.22)	•Thomsen et al. 2022[39]<br>•Goodman et al. 2003[40]<br>•Guo et al. 2004[6]	•Motor vehicle mechanics<br>•Motor vehicle mechanics (cohort studies)<br>•Car mechanics exposed to Diesel exhaust
2310	Manufacture of glass and glass products	Glass production and processing workers	Crystalline silica<br>Asbestos (various forms)<br>Chromium IV and chromium compounds<br>Nichel and nichel compounds<br>Cadmium and cadmium compounds	•NA			•RR 2.18 (95% CI 1.44-3.29)<br>•SMR 1.23 (90% CI 1.00-1.51)<br>•SMR 2.09 (95% CI 1.24-3.32)	•Crosignani et al. 2006[4]<br>•Pirastu et al. 1998[42]<br>•Cordioli et al. 1987[43]	•Glass production workers<br>•Glass production workers<br>•Glass production workers			

DEE: Diesel engine exhaust, EC: Elemental carbon, SD: Standard deviation, B: benzene, Asb: asbestos, PM: particulate matter, BTEX: benzene, toluene, ethylbenzene, xylenes, cPAH: carcinogenic polycyclic aromatic hydrocarbons, CO: carbon monoxide, VOCs: volatile organic compounds, CS: crystalline silica, WF: welding fumes, CHVI: hexavalent chromium,

Supplementary References

1. Davis, M. E. *et al.* Driver exposure to combustion particles in the U.S. Trucking industry. *J Occup Environ Hyg* **4**, 848–854 (2007).

2. Boffetta, P., Cherrie, J., Hughson, G. & Pitard, A. Cancer risk from diesel emissions exposure in Central and Eastern Europe: a feasibility study. in *Research Directions to Improve Estimates of Human Exposure and Risk from Diesel Exhaust* (ed. McLellan, G.) 57–78 (Health Effects Institute, Boston, MA, 2002).

3. Davies, H. *et al.* Mutations of the BRAF gene in human cancer. *Nature* **417**, 949–954 (2002).

4. Crosignani, P. *et al.* The Italian surveillance system for occupational cancers: Characteristics, initial results, and future prospects. *American J Industrial Med* **49**, 791–798 (2006).

5. Lagorio, S., Forastiere, F., Rapiti, E., Di Pietro, A. & Costa, G. [Economic and occupational activities at an increased risk of mortality for lung tumors in Turin (1981-89) and in Italy (1981-82)]. *Med Lav* **86**, 309–324 (1995).

6. Guo, J. *et al.* Occupational exposure to diesel and gasoline engine exhausts and risk of lung cancer among Finnish workers. *American J Industrial Med* **45**, 483–490 (2004).

7. Cattaneo, A. *et al.* Personal exposure of traffic police officers to particulate matter, carbon monoxide, and benzene in the city of Milan, Italy. *J Occup Environ Hyg* **7**, 342–351 (2010).

8. Zahm, S. H., Brownson, R. C., Chang, J. C. & Davis, J. R. Study of lung cancer histologic types, occupation, and smoking in missouri. *American J Industrial Med* **15**, 565–578 (1989).

9. Chen, J. *et al.* Long-term exposure to ambient air pollution and risk of lung cancer – A comparative analysis of incidence and mortality in four administrative cohorts in the ELAPSE study. *Environmental Research* **263**, 120236 (2024).

10. Paik, N. W., Walcott, R. J. & Brogan, P. A. Worker exposure to asbestos during removal of sprayed material and renovation activity in buildings containing sprayed material. *Am Ind Hyg Assoc J* **44**, 428–432 (1983).

11. Richiardi, L. *et al.* Occupational risk factors for lung cancer in men and women: a population-based case-control study in Italy. *Cancer Causes Control* **15**, 285–294 (2004).

12. Scarselli, A., Binazzi, A., Forastiere, F., Cavariani, F. & Marinaccio, A. Industry and job-specific mortality after occupational exposure to silica dust. *Occup Med (Lond)* **61**, 422–429 (2011).

13. CONSAD Research Corporation. *Economic Analysis of the Proposed Revisions to the OSHA Asbestos Standards for Construction and General Industry*. (1990).

14. Paci, E., Buiatti, E. & Geddes, M. A case-referent study of lung tumors in non-asbestos textile workers. *Am J Ind Med* **11**, 267–273 (1987).

15. Ietri, E. *et al.* Cohort mortality study of rubber and plastics product makers in Italy. *Occup Med* **47**, 417–422 (1997).

16. d'Errico, A. *et al.* [Occupational mortality in Italy during 1992, assessed through record-linkage between pension records and death certificates]. *Med Lav* **96 Suppl**, s52-65 (2005).

17. Plato, N., Lewné, M. & Gustavsson, P. A historical job-exposure matrix for occupational exposure to diesel exhaust using elemental carbon as an indicator of exposure. *Archives of Environmental & Occupational Health* **75**, 321–332 (2020).

18. Briancesco, R., Alaimo, C. & Bonanni, E. Cancer risk among farmers in the Province of Vercelli (Italy) from 2002 to 2005: an ecological study. *Annali di Igiene Medicina Preventiva e di Comunità* 255–263 (2014) doi:10.7416/ai.2014.1983.

19. Gianicolo, E. A. L. *et al.* Long-term effect of arsenic exposure: Results from an occupational cohort study. *American J Industrial Med* **62**, 145–155 (2019).

20. Neri, F. *et al.* Determining exhaust fumes exposure in chainsaw operations. *Environmental Pollution* **218**, 1162–1169 (2016).

21. D'Antonio, P., Toscano, F., Moretti, N., De Iorio, N. & Fiorentino, C. Analysis of Chainsaw Emissions during Chestnut Wood Operations and Their Health Implications. *Applied Sciences* **14**, 2496 (2024).

22. Lewné, M., Plato, N. & Gustavsson, P. Exposure to Particles, Elemental Carbon and Nitrogen Dioxide in Workers Exposed to Motor Exhaust. *The Annals of Occupational Hygiene* (2007) doi:10.1093/annhyg/mem046.

23. Consonni, D. *et al.* Increased lung cancer risk among bricklayers in an Italian population-based case–control study. *American J Industrial Med* **55**, 423–428 (2012).

24. INAIL & FORMEDIL. *L'esposizione a Silice Cristallina Respirabile Nei Cantieri Edili e Di Ingegneria Civile.* https://www.inail.it/cs/internet/docs/alg-pubbl-silice-cristallina-respirabile-cantieri-edili-2024.pdf (2024).

25. Massari, S. *et al.* Cancer mortality and sectors of employment: a cohort study in Italy. *BMC Public Health* **25**, 458 (2025).

26. Consonni, D. *et al.* Lung Cancer and Occupation in a Population-based Case-Control Study. *American Journal of Epidemiology* **171**, 323–333 (2010).

27. Gerosa, A., Scarnato, C., Marchesini, B., Ietri, E. & Pavone, V. L. M. Studio di mortalità in una coorte di esposti nella lavorazione a caldo di materie plastiche e gomma. *E&P* **41**, 125–133 (2017).

28. Mundt, K. A., Dell, L. D., Crawford, L., Sax, S. N. & Boffetta, P. Cancer Risk Associated With Exposure to Bitumen and Bitumen Fumes: An Updated Systematic Review and Meta-Analysis. *Journal of Occupational & Environmental Medicine* **60**, e6–e54 (2018).

29. Lombardy Region. *Handbook for Improving the Health and Safety Conditions of Workers in Metal Welding Activities*. https://www.inail.it/cs/internet/docs/alg-pubbl-vademecum-saldatura-metalli.pdf (2012).

30. Kvam, B. M., Romundstad, P. R., Boffetta, P. & Andersen, A. Cancer in the Norwegian printing industry. *Scand J Work Environ Health* **31**, 36–43 (2005).

31. Luce, D. *et al.* Cancer mortality among magazine printing workers. *Occup Environ Med* **54**, 264–267 (1997).

32. Verma, D. K. *et al.* A comparison of sampling and analytical methods for assessing occupational exposure to diesel exhaust in a railroad work environment. *Appl Occup Environ Hyg* **14**, 701–714 (1999).

33. Crump, K. S. LUNG CANCER MORTALITY AND DIESEL EXHAUST: REANALYSIS OF A RETROSPECTIVE COHORT STUDY OF U.S. RAILROAD WORKERS. *Inhalation Toxicology* **11**, 1–17 (1999).

34. Seshagiri, B. Exposure to diesel exhaust emissions on board locomotives. *AIHA J (Fairfax, Va)* **64**, 678–683 (2003).

35. Tessari, R., Canova, C. & Simonato, L. [Epidemiological investigation on the health status of employees in two factories manufacturing and repairing railway rolling stock: a historical perspective study of mortality]. *Med Lav* **95**, 381–391 (2004).

36. Verdel, U., Iotti, A. & Castelletti Ballarà, G. *Historical Mapping of Asbestos Exposure in Italian Industry: Assessment of Asbestos Exposure for the Purpose of Social Security Benefits*. https://onanotiziarioamianto.it/wp-content/uploads/2020/10/ArtVERDELIOTTI-5.pdf (1997).

37. Region of Tuscany. *Regional Plan for Asbestos*. https://www.regione.toscana.it/documents/10180/11840104/Delibera_102_1997_Piano_Regionale_Amianto.pdf (1997).

38. Felten, M. K. *et al.* Retrospective exposure assessment to airborne asbestos among power industry workers. *J Occup Med Toxicol* **5**, 15 (2010).

39. Thomsen, R. W. *et al.* Risk of asbestosis, mesothelioma, other lung disease or death among motor vehicle mechanics: a 45-year Danish cohort study. *Thorax* **77**, 477–485 (2022).

40. Goodman, M. *et al.* Mesothelioma and lung cancer among motor vehicle mechanics: a meta-analysis. *Ann Occup Hyg* **48**, 309–326 (2004).

41. Groves, J. & Cain, J. R. A survey of exposure to diesel engine exhaust emissions in the workplace. *Ann Occup Hyg* **44**, 435–447 (2000).

42. Pirastu, R. *et al.* Cancer mortality of art glass workers in Tuscany, Italy. *Scand J Work Environ Health* **24**, 386–391 (1998).

43. Cordioli, G. *et al.* [Tumor mortality in a cohort of glass industry workers]. *Epidemiol Prev* **9**, 16–18 (1987).
