# Supplementary material for: Exposure to Occupational Carcinogens and Non-Oncogene Addicted Phenotype in Lung Cancer: Results from a Real-Life Observational Study
Source: Cancers (Basel). 2025 Sep 13;17(18):2997. doi: 10.3390/cancers17182997 (PMC12468263; doi:10.3390/cancers17182997)
Supplement: Supplementary file 1 [file cancers-17-02997-s001.zip › Table S11.pdf]

Table S11. Job tasks and duration, and main exposure of workers classified as high exposed. Pavia-Milan (2021-2023).

n	ISIC description	Job task	Job duration (years)	Timespan	Main exposure	Note
1	Freight transport by road	Driver and basic maintenance mechanic of trucks	35	1986-2021	Diesel exhaust	
2	Public order and safety activities	Urban police officer with ongoing responsibilities for traffic management and direction	39	1977-2016	Diesel exhaust	
3	Electrical, plumbing and other construction installation activities	Plumber and thermal systems installer/technician	40	1979-2019	Asbestos (various forms)<br>Crystalline silica<br>Combustion-derived soot<br>Welding fumes	
4	Manufacture of man-made fibres	Electrical technician in a rayon manufacturing plant	22	1974-1996	Asbestos (various forms)	Electrian all along his work history (1950-1996)
5	Freight transport by road	Driver and basic maintenance mechanic of trucks	30	1989-2020	Diesel exhaust	
6	Manufacture of plastics products	Worker engaged in the production of plastic forniture	18	1970-1988	Pigments for plastic products manufacture	Another long work period spent as farmer
7	Freight transport by road	Driver and basic maintenance mechanic of trucks	38	1978-2016	Diesel exhaust	
8	Growing of non-perennial crops	Driver and basic maintenance of tractors and other agricoltural machines<br>Soil preparation and tillage; subsequent operations (fertilization, sowing, etc.); harvesting and primary processing of the crop	56	1965-2021	Diesel exhaust<br>Arsenic inorganic compounds<br>Crystalline silica<br>Combustion-derived soot	
9	Silviculture and other forestry activities	Use of chainsaw or other tools for tree felling or cutting, stump removal and root grubbing	37	1973-2010	Diesel exhaust	
10	Construction of buildings	Bricklayer	51	1956-2007	Asbestos (various forms)<br>Crystalline silica<br>Combustion-derived soot<br>Lead chromate<br>Pitch, coal tar<br>Welding fumes	
11	Growing of non-perennial crops	Farmer, specifically involved in corn cultivation; Driver and basic maintenance of tractors	24	1993-2017	Diesel exhaust<br>Arsenic inorganic compounds<br>Combustion-derived soot	

12	Construction of buildings	Bricklayer and worker involved in window and door frame installation	43	1956-1999	Asbestos (various forms)<br>Crystalline silica<br>Combustion-derived soot<br>Lead chromate<br>Pitch, coal tar<br>Welding fumes	
13	Transport via pipeline	Fuel station operator and attendant selling gasoline and diesel	20	2000-2020	Diesel exhaust	
14	Public order and safety activities	Urban police officer with ongoing responsibilities for traffic management and direction	30	1975-2005	Diesel exhaust	
15	Manufacture of rubber tires and tubes	Machine press and mixing operator involved in tire production	10	1968-1978	Bis(chloromethyl) ether<br>Pigments for rubber production	
16	Growing of non-perennial crops	Driver and basic maintenance of tractors and other agricoltural machines	34	1960-1994	Diesel exhaust<br>Arsenic inorganic compounds<br>Combustion-derived soot	
17	Manufacture of domestic appliances	Worker involved in the production and assembly of household appliances	22	1970-1993	Welding fumes<br>Crystalline silica	
18	Construction of roads and railways	Excavator machine operators and workers involved in road asphalting and surfacing	50	1958-2008	PAH<br>Diesel exhaust<br>Asbestos (various forms)<br>Crystalline silica<br>Pitch, coal tar	
19	Growing of non-perennial crops	Farmer; Applicator of Parasite control products for animals and pesticides	23	1982-2005	Arsenic inorganic compounds<br>Chromium compounds	
20	Freight transport by road	Driver and basic maintenance mechanic of trucks	23	1990-2013	Diesel exhaust	Another long work period spent as tower crane operator in civil construction
21	Casting of iron and steel	Metal foundry worker	18	1970-1988	Crystalline silica<br>Chromium IV and chromium compounds<br>Nichel and nichel compounds	
22	Manufacture of other fabricated metal products; metalworking service activities	Mechanical and metalworking industry worker	14	1988-2001	Welding fumes<br>Chromium IV and chromium compounds<br>Nichel and nichel compounds	Another period with the same job task in 1967-1978
23	Construction of buildings	Bricklayer and building painter	48	1974-2022	Asbestos (various forms)<br>Crystalline silica<br>Combustion-derived soot<br>Lead chromate<br>Pitch, coal tar<br>Welding fumes	

24	Printing and service activities related to printing	Worker specialized in lithography and engraving	43	1973-2016	Chromium IV and chromium compounds<br>Nichel and nichel compounds
25	Electrical, plumbing and other construction installation activities	Plumber and thermal systems installer/technician	42	1977-2019	Asbestos (various forms)<br>Crystalline silica<br>Combustion-derived soot<br>Welding fumes
26	Freight transport by road	Professional car driver and maintenance mechanic	38	1972-2010	Diesel exhaust
27	Electrical, plumbing and other construction installation activities	Plumber and thermal systems installer/technician	40	1971-2011	Asbestos (various forms)<br>Crystalline silica<br>Combustion-derived soot<br>Welding fumes
28	Construction of buildings	Bricklayer and carpenter	20	1984-2004	Asbestos (various forms)<br>Crystalline silica<br>Combustion-derived soot<br>Welding fumes
29	Construction of roads and railways	Excavator machine operators and workers involved in road asphalting and surfacing	31	1958-2003	PAH<br>Diesel exhaust<br>Asbestos (various forms)<br>Crystalline silica<br>Pitch, coal tar
30	Construction of buildings	Bricklayer also employed in finishing and completion works (plasterer/stucco worker)	53	1959-2012	Asbestos (various forms)<br>Crystalline silica<br>Combustion-derived soot<br>Welding fumes
31	Manufacture of domestic appliances	Worker involved in refrigerator manufacturing	25	1963-1989	Welding fumes<br>Crystalline silica
32	Freight transport by road	Driver and basic maintenance mechanic of trucks	43	1974-2017	Diesel exhaust
33	Freight transport by road	Driver and basic maintenance mechanic of trucks	38	1970-2008	Diesel exhaust
34	Service activities incidental to land transportation	Maintenance technician and sector supervisor in a railway company	26	1975-2001	Welding fumes<br>Diesel exhaust<br>Asbestos (various form)
35	Freight rail transport	Railway worker, train operator	34	1977-2011	Diesel exhaust

36	Freight rail transport	Railway worker, track inspection officer	35	1988-2023	Diesel exhaust	
37	Construction of buildings	Bricklayer and ceramic flooring specialist	20	1985-2005	Asbestos (various forms)<br>Crystalline silica<br>Combustion-derived soot<br>Pitch, coal tar<br>Welding fumes	
38	Construction of buildings	Bricklayer and skilled structural carpenter	20	1995-2015	Crystalline silica<br>Combustion-derived soot<br>Pitch, coal tar<br>Welding fumes	Also a previous work period as a carpenter in another construction company (1980-1995)
39	Electric power generation, transmission and distribution	Initially employed as an operator, and subsequently promoted to supervisor responsible for plant operation and power generation management in thermal power stations	22	1978-2000	Asbestos (various forms)	
40	Maintenance and repair of motor vehicles	Motor vehicle mechanic	28	1972-2000	Diesel exhaust<br>Welding fumes<br>Asbestos (various forms)	
41	Construction of buildings	Construction worker on scaffolding	49	1973-2022	Asbestos (various forms)<br>Crystalline silica<br>Combustion-derived soot<br>Pitch, coal tar<br>Welding fumes	
42	Growing of non-perennial crops	Farmer, specifically involved in corn cultivation; Driver and basic maintenance of tractors	58	1964-2022	Diesel exhaust<br>Arsenic inorganic compounds<br>Combustion-derived soot	
43	Maintenance and repair of motor vehicles	Automotive body technician and refinisher	40	1978-2018	Welding fumes<br>Diesel exhaust	Another period with the same job task in 1963-1972
44	Printing and service activities related to printing	Worker specialized in lithography and engraving	19	1973-1992	Chromium IV and chromium compounds<br>Nichel and nichel compounds	
45	Freight transport by road	Warehouse worker, then driver and basic maintenance mechanic of trucks	43	1972-2015	Diesel exhaust	
46	Manufacture of rubber tyres and tubes	Tire production worker	28	1974-2002	Bis(chloromethyl) ether<br>Pigments for rubber production	
47	Manufacture of glass and glass products	Glassblowing worker in a glass manufacturing plant	12	1968-1980	Arsenic inorganic compounds<br>Chromium compounds<br>Crystalline silica	
